# Invasive group A *Streptococcus* disease in French-Canadian children is not associated with a defect in MyD88/IRAK4-pathway

**DOI:** 10.1186/1710-1492-10-9

**Published:** 2014-02-05

**Authors:** Isabel Fernandez, Rose-Marie Brito, Philippe Bidet, Fabien Rallu, Celine Laferrière, Philippe Ovetchkine, Francoise Le Deist

**Affiliations:** 1 Department of Microbiology, Infectiology and Immunology, CHU Sainte-Justine and University of Montreal, Montreal (Quebec), Canada; 2 Department of Microbiology and Immunology, CHU Sainte-Justine, Montreal (Quebec), Canada; 3 CHU Sainte-Justine Research Center, University of Montreal, Montreal (Quebec), Canada; 4 Laboratory of Microbiology, Hospital Robert-Debré, Assistance Publique-Hôpitaux de Paris (APHP) and University Paris Diderot, Sorbonne Paris Cité, EA 3105 Paris, France; 5 Department of Paediatrics, CHU Sainte-Justine and University of Montreal, Montreal (Quebec), Canada

**Keywords:** Invasive group A *Streptococcus*, MyD88/IRAK4-mediated signalling, Innate immunity

## Abstract

**Background:**

Beta-hemolytic Group A Streptococcus invasive disease (iGASd) has been subject to intense research since its re-emergence in the late 1980s. In Quebec, an increase in the number of severe iGASd cases has recently been observed. Because of the inter-individual variability in the severity of iGASd, a hereditary predisposition to invasive disease can be suspected. Given that iGASd occurs in MyD88- and IRAK4-deficient patients, although rarely, the increasing frequency of iGASd in the population of French-Canadian children may be associated with a deficiency in the host’s innate immune response.

**Methods:**

In this report, we assessed the influence of: (i) bacterial genotype and virulence factors, (ii) immune-cellular features, and (iii) Myd88/IRAK4-dependent response to GAS in vitro on the susceptibility to iGASd in a paediatric cohort of 16 children: 11 French-Canadian and 5 from diverse origin.

**Findings:**

GAS virulence factors and genotype are not implicated in the susceptibility toward iGASd, and cellular and MyD88/IRAK4 deficiencies are excluded in our patients.

**Conclusions:**

Although it has been shown that the MyD88/IRAK4-dependent signal is involved in the response to invasive GAS, our data indicates that a MyD88/IRAK4-mediated signalling defect is not the main factor responsible for the susceptibility to severe iGASd in a paediatric population from the province of Quebec.

## Background

Beta-hemolytic Group A Streptococcus (GAS, *Streptococcus pyogenes*) is a Gram-positive bacteria responsible for a variety of human infections, ranging from pharyngitis to invasive infections. Since the 1980s, an important resurgence of invasive GAS diseases (iGASd), defined as an illness associated with isolation of GAS from a normally sterile site, has been noted [1]. For instance, in Quebec and particularly Montreal, an unusually large number of iGASd cases has been reported from 1995 to 2001 (2.4 iGASd cases per 100.000 habitants), with a mortality rate of 14 % and a significant increase in pneumonia (from 0.06 to 0.50 per 100.000 habitants) [1], From January 2009 to January 2011, 596 cases of severe iGASd were noted in the “Registre central des maladies à déclaration obligatoire of Quebec”, 137 of them in Montreal. (See, "Surveillance épidémiologique rehaussée des infections invasives à streptocoque du groupe A dans la province de Québec, Bilan du 18 janvier 2009 au 17 janvier 2011"). Invasive GASd includes different pathologies such as severe necrotizing fasciitis, cellulites with bacteraemia, osteoarthritis and pleural empyema. The variability of individual responses to GAS infection represents an intriguing model for investigating and understanding the role of host-pathogen interactions in the outcome of an invasive infectious disease. To date, some identified risk factors for iGASd in paediatrics are young age, varicella, and bacterial virulence factors [2]. The inability to control bacterial burden during primary GAS infection might result from an absence or inefficient inflammatory response, or from an overreaction of the response. Susceptibility to iGASd is characterized by an impaired bacterial clearance and the response triggered by the same virulence factor varies individually and correlates with the severity of iGASd [3,4].

In humans, MyD88/IRAK-4 deficiency is associated with life threatening, invasive, pyogenic bacterial diseases, typically caused by Gram-positive species, mainly *Streptococcus pneumoniae* and *Staphylococcus aureus*[5,6]. Patients with IRAK4/MyD88 deficiency have generally normal basic immunologic evaluation (T, B, and NK cell count and humoral assessment including serum levels of the IgM, IgA, IgG, and IgG subclasses and normal vaccines responses), making their diagnosis a real challenge. Invasive GASd occurs in some MyD88/IRAK4-deficient individuals, although not extensively and, in our knowledge, rarely as the only pathogen [6].

Several regions in the province of Quebec have been studied extensively for their founder effect and their increased prevalence of hereditary diseases with an impact in medical genetics [7]. Interestingly, the increased incidence of iGASd in healthy children suggests that the host’s genetics may play a part in the predisposition to invasive disease. Therefore, the French-Canadian origin of our cohort and a putative founder effect may favor the establishment of a unique hereditary predisposition to iGASd in the French-Canadian population. Our main objective was to define a putative defect of innate immune signalling underlying the increase susceptibility to iGASd in Quebec children.

## Methods and Findings

In this report, we assessed Myd88/IRAK4-dependent response to GAS in vitro in a paediatric cohort of 16 children: 11 French-Canadian and 5 from diverse origin, and well 15 healthy age-matched individuals. No consanguinity in parents/grand-parents was reported for any of the 16 patients. Patients had no history of varicella preceding iGASd. A standardized form was used to, in retrospect, collect ancestral (including a genogram) and demographic information, previous medical conditions, clinical diagnosis criteria, and biologic and microbiologic findings. No records were available for data on humoral immunity, but absence of severe infections before and after iGASd could indicate an adequate humoral immunity. The characteristics of the patients and of the isolated streptococcal strains are summarized in Table 1.

**Table 1:** Characteristics of iGAS patients and streptococcal strains

**Patient number**	**Age at iGASd (years)**	**Delay between assay and iGASd (years)**	**iGASd manifestations**	***emm*-type**	**Virulence factors gene profile**
**1**	1.6	0.5	Necrotizing fasciitis/osteomyelitis	12	*spe*A, *spe*B*, spe*C, *ssa*
**2**	3.1	1.6	pneumonia with empyema	1	*spe*A, *spe*B*, sme*Z-1, *sic*
**3**	5.1	1.4	bacteremia	1	*spe*A, *spe*B*, sme*Z-1, *sic*
**4**	6.2	1.4	Cellulites with bacteremia	1	*spe*A, *spe*B*, sme*Z-1, *sic*
**5**	10.3	0.1	osteomyelitis	12	*spe*B*, spe*C, *ssa*
**6**	7.0	0.1	bacteremia	12	*spe*B*, spe*C, *ssa*
**7**	3.4	0.2	pneumonia with empyema	1	*spe*B*, ssa*, *sme*Z-1, *sic*
**8**	16.2	3.4	necrotizing fasciitis	5	*spe*B*. spe*C
**9**	9.4	3.2	necrotizing fasciitis	4	*spe*B*, spe*C, *ssa*, *sme*Z-1
**10**	8.8	0.2	bacteremia	1	*spe*B, *ssa*, *sme*Z-1
**11**	1.8	1.7	bacteremia	4	*spe*B*, spe*C, *ssa*, *sme*Z-1
**12**	3.8	0.2	pneumonia with empyema	nd	nd
**13**	4.6	4.5	meningitis	89	*spe*B*, spe*C
**14**	1.9	4.0	bacteremia	49	*spe*B
**15**	6.1	3.9	arthritis	4	*spe*C, *ssa*, *sme*Z-1
**16**	2.5	4.7	arthritis	12	*spe*B
**Median**	4.8	1.5			
**Range**	1.6-16.2	0.1-4.5			

iGASd = invasive Group A Streptococcal disease. nd: not done. iGASd = invasive Group A Streptococcal disease. nd: not done. French-Canadian: P1, P2, P3, P4, P6, P7, P10, P11, P13, P14, P15.

Bacterial M protein encoded by the *emm* gene is an important epidemiologic marker. Bacterial isolates were thus characterized by the *emm* type and toxin gene profiling, as previously described [2] (Table 1). Presence of streptococcal pyrogenic exotoxin genes A, B, and C (*speA*, *speB*, *spec*) streptococcal mitogenic exotoxin Z gene allele 1 (*smeZ*-1), and streptococcal inhibitor of complement-mediated lysis gene (*sic*) were detected as previously described [2]. The *emm* sequencing revealed heterogeneity among the isolates. However, there is no correlation between clinical presentation, bacterial genotype and/or the studied virulence factors.

To discard an underlying cellular immune-defect in the patients, a quantitative evaluation of immune cell populations was performed: enumeration and characterization of T and B lymphocytes and NK and TcRγδ T cell counts (Additional file 1: Table S1 and S2). Except for the two patients with a slight lymphopenia (P3 and P11), no immune abnormalities were noticed in the cell populations. The excess of CD3^+^CD4^-^CD8^-^ T cell population in seven patients (P2, P3, P4, P5, P7, P9 and P10) can be explained by an excess of TcRγδ T lymphocytes (as found in P1 and P7). In conclusion, no indication of cellular immunodeficiency was observed in iGASd children.

**Figure 1:**
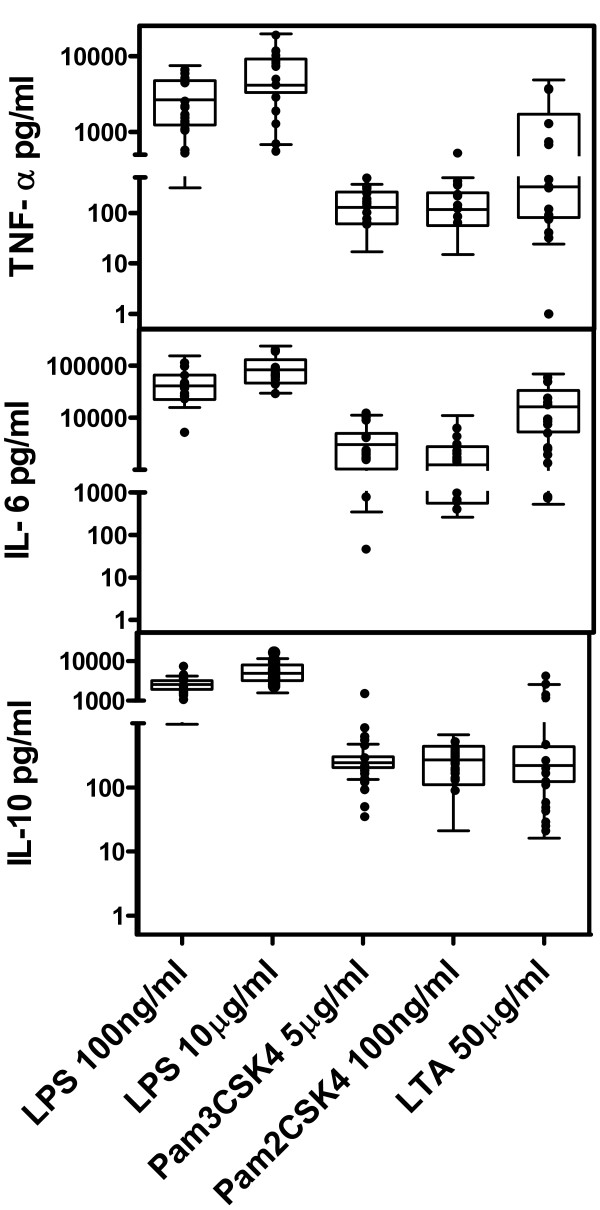
**In vitro secretion of cytokines (TNF-α, IL-6, and IL-10) after 24 h of TLR-stimulation of whole blood (1/5 dilution).** Results in patients (●) are expressed as mean of secretion calculated as (secretion in activated -secretion in non stimulated cells) from duplicate results. Not statistically significant differences between healthy individuals and patients were found (Mann–Whitney non-parametric test). Box: 5–95% percentile; Whiskers: Range; ▬ median in healthy controls.

To assess a hypothetical MyD88/IRAk4 signalling defect, a functional study was performed after Toll-like receptor (TLR)-4 (LPS from *Salmonella enterica*) and TLR-2 (LTA from S*accharomyces Cerevisia*), TLR-2/TLR-6 heterodimers (lipopeptide Pam2CSK4), and TLR-2/TLR-1 heterodimers (lipopeptide Pam3CSK4) activation in presence of polymyxin B, as previously described [6]. After 1 h incubation, CD62L cleavage on granulocytes was evaluated by flow cytometry (FACScanto cytometer, Becton Dickinson) and after 24 h, IL-6, TNF-α, and IL-10 secretion was assessed by ELISA. Intriguingly, no defective TLR-dependent response was observed for CD62L cleavage (Additional file 1: Table S3), nor in cytokine secretion (Figure 1) (Mann–Whitney non-parametric test).

## Discussion

The fact that MyD88-/IRAK4-deficient patients suffer from Gram-positive bacteria infections pointed to a defect on the innate immunity signal in iGASd. The major limitation to this study is the small number of subjects involved. However, the French-Canadian origin of 11 patients can imply a hereditary predisposition to iGASd susceptibility, even in a limited cohort (the 2600 settlers in Nouvelle-France before 1680 contributed about two thirds of the gene pool of the current French-Canadian population) [7]. However, our cohort of French-Canadian patients seems to be heterogeneous and their susceptibility to invasive infection is not likely related to a defect on MyD88/IRAK4 signalling pathways.

Several, in vitro studies underlined the importance of MyD88 signalling in the response to GAS. For instance, mouse dendritic cell maturation and cytokine production in response to GAS is entirely MyD88-dependent, indicating the important role of the innate immunity in streptococcal infection [8,9]. The specific receptor capable of recognizing GAS by mouse dendritic cells is still unidentified. Alternatively, Gratz et al. [10] demonstrated that GAS can induce type I IFN production in mouse macrophages in a MyD88-dependent and a MyD88-independent manner ^10^. Our results are in agreement with this study and we cannot exclude a defect in a Myd88-independent signalling pathway in iGASd patients.

## Conclusion

MyD88/IRAK-4 deficiencies are excluded in an immunocompetent French-Canadian cohort of children with severe beta haemolytic group A Streptococcus infection. The existence of an unknown receptor upstream of MyD88 implicated in GAS recognition and/or a defect in innate immune response in non-haematopoietic cells as epithelial cells, and/or a complex stimulation of multiple signalling pathways for an efficient immune defence against iGASd should be considered.

## Abbreviations

GAS: Beta-hemolytic group A streptococcus; iGASd: invasive GAS disease; TLR: Toll-like receptor.

## Competing interests

Authors have no financial relationships relevant to this article to disclose and no conflicts of interest. The manuscript has not been previously published, is not under consideration elsewhere and will not be submitted elsewhere while under consideration by Allergy, Asthma and Clinical Immunology.

## Authors’ contributions

All authors are responsible for the reported research and have participated in the concept and design, analysis and interpretation of data, drafting or revising of the manuscript, and have approved the manuscript as submitted. IMF coordinated data collection, drafted the manuscript, and approved the final manuscript as submitted. RMB, PB, FR, and CL carried out analyses, reviewed the manuscript, and approved the final manuscript as submitted. PO and FLD conceptualized and designed the study, critically reviewed the manuscript, and approved the final manuscript as submitted. The manuscript has been read and approved by all authors.

## Supplementary Material

Additional file 1: Table S1 Lymphocyte populations. **Table S2.** Subpopulations of T and B lymphocytes. **Table S3.** TLR-dependent cleavage of CD62L* (%). Click here for file
