# Supplementary material for: Invasive group A Streptococcus disease in French-Canadian children is not associated with a defect in MyD88/IRAK4-pathway
Source: Allergy Asthma Clin Immunol. 2014 Feb 5;10(1):9. doi: 10.1186/1710-1492-10-9 (PMC3927219; doi:10.1186/1710-1492-10-9)
Supplement: Additional file 1: Table S1 — Lymphocyte populations. Table S2. Subpopulations of T and B lymphocytes. Table S3. TLR-dependent cleavage of CD62L* (%). [file 1710-1492-10-9-S1.doc]

**Supplementary Table 1: Lymphocyte populations**

| **Patient**  **number** | **CD3+**  **/mm^3^** | **CD4+**  **/mm^3^** | **CD8+**  **/mm^3^** | **CD19+**  **/mm^3^** | **CD3-CD56+**  **/mm^3^** |
| --- | --- | --- | --- | --- | --- |
| **1** | **6534**  (2220-3835)* | **3663**  (1360-2500) | **2574**  (580-1224) | 1584  (590-1450) | **1584**  (147-532) |
| **2** | 2504  (1630-3180) | 1078  (900-2030) | 729  (460-1000) | 349  (360-820) | 114  (95-455) |
| **3** | 1104  (1370-2555) | **552**  (735-1550) | 276  (380-830) | 294  (266-590) | 368  (130-330) |
| **4** | 2188  (1370-2555) | 831  (735-1550) | 609  (380-830) | 332  (266-590) | 222  (130-330) |
| **5** | 1508  (1340-2110) | 702  (695-1320) | 416  (365-640) | 390  (225-500) | **663**  (100-360) |
| **6** | 2263  (1705-3555) | 1426  (950-2255) | 527  (505-1115) | 403  (415-1215) | 211  (120-485) |
| **7** | 2022  (1705-3555) | 886  (950-2255) | 609  (505-1115) | 471  (415-1215) | 141  (120-485) |
| **8** | 1142  (1061-2283) | 709  (555-1395) | 294  (290-830) | 190  (110-405) | 301  (85-345) |
| **9** | 1714  (1215-1855) | 1214  (630-1155) | **167**  (317-622) | 333  (180-460) | 252  (80-250) |
| **10** | 2046  (1340-2110) | 713  (695-1320) | 496  (365-640) | 372  (225-500) | 434  (100-360) |
| **11** | **1229**  (1705-3555) | **605**  (950-2255) | 468  (505-1115) | 371  (415-1215) | 246  (120-485) |
| **12** | 3184  (1630-3180) | 1886  (900-2030) | 964  (460-1000) | 629  (360-820) | 189  (95-455) |
| **13** | 1981  (1340-2110) | 1367  (695-1320) | 502  (365-640) | 391  (225-500) | 265  (100-360) |
| **14** | 1733  (1370-2555) | 1026  (735-1550) | 524  (380-830) | 342  (266-590) | 103  (130-330) |
| **15** | 2225  (1340-2110) | 1156  (695-1320) | 867  (365-640) | 462  (225-500) | 139  (100-360) |
| **16** | **3624**  (1370-2555) | **2129**  (735-1550) | **1087**  (380-830) | 634  (266-590) | 136  (130-330) |

*Age-matched normal values (ranged from 10^th^ to 90^th^ percentiles) are indicated in brackets. P1 and P16 presented a minor hyper-lymphocytosis, and P3 and P11 a slight lymphopenia for CD3+ CD4+ T lymphocytes. For the rest, no significant immune anomalies in cell populations were noticed.

**Supplementary Table 2: Subpopulations of T and B lymphocytes**

| **Patient**  **number** | **TcRγδ**  **/CD3 %[/mm^3^]*** | **CD31+CD45RA+**  **/CD4 % (normal value)^§^** | **CD27/CD19%**  **(normal value)^§^** |
| --- | --- | --- | --- |
| 1 | 7 [457] | 61 (53-70) | 10 (7-19) |
| 2 | ND | 63 (48-68) | 25 (12-28) |
| 3 | ND | 45 (47-65) | 23 (12-28) |
| 4 | ND | 55 (47-65) | 15 (12-28) |
| 5 | ND | 56 (44-60) | 15 (12-28) |
| 6 | ND | 61 (50-70) | 14 (10-20) |
| 7 | 21 [425] | 68 (50-70) | 12 (10-20) |
| 8 | ND | 33 (22-60) | 17 (11-37) |
| 9 | ND | 58 (35-60) | 13 (11-24) |
| 10 | ND | 33 (44-60) | ND |
| 11 | ND | 44 (50-70) | 20 (10-20) |
| 12 | 7 [223] | 58 (48-68) | 17 (12-28) |
| 13 | 4 [79] | 67 (44-60) | 23 (12-28) |
| 14 | 8 [139] | 46 (47-65) | 28 (12-28) |
| 15 | 8 [178] | 51 (44-60) | 39 (12-28) |
| 16 | 5 [181] | 70 (47-65) | 10 (12-28) |

* normal value: TcRγδ/CD3 % 2< N <10 or TcRγδ/mm^3^ or 25<N<300

**^§^** age-matched normal value. ND Not done.

**Supplementary Table 3: TLR-dependent cleavage of CD62L* (%)**

**CONTROLS PATIENTS**

**Activator Mean S.D Mean S.D**

LPS 10ng/ml 94.4 2.8 91.7 13.0

Zymosan 50μg/ml 94.3 4.8 94.2 7.8

Pam3CSK4 1μg/ml 88.3 13.0 84.8 20.1

Pam2CSK4 10ng/ml 86.8 11.1 86.4 1.0

Pam2CSK4 100ng/ml 86.6 12.8 89.7 4.8

LTA 50μg/ml 94.4 7.9 92.8 10.5

TNF 1μg/ml 96.2 1.9 95.3 2.1

PMA 2μg/ml 94.2 8.1 94.6 5.0

*: Calculated as (mean fluorescence without activator – mean fluorescence with activator)/(mean fluorescence without activator – non specific mean fluorescence) x 100.
